# Lipopolysaccharide-Binding Protein Downregulates Fractalkine through Activation of p38 MAPK and NF-*κ*B

**DOI:** 10.1155/2017/9734837

**Published:** 2017-05-29

**Authors:** Xia Huang, Yi Zeng, Yujie Jiang, Yueqiu Qin, Weigui Luo, Shulin Xiang, Suren R. Sooranna, Liao Pinhu

**Affiliations:** ^1^The First Clinical Medical College of Jinan University, Guangzhou, Guangdong Province 510630, China; ^2^Department of Respiratory Medicine, Youjiang Medical University for Nationalities, Baise, Guangxi Zhuang Autonomous Region 533000, China; ^3^Department of Central Laboratory, Youjiang Medical University for Nationalities, Baise, Guangxi Zhuang Autonomous Region 533000, China; ^4^Department of Digestive Medicine, Youjiang Medical University for Nationalities, Baise, Guangxi Zhuang Autonomous Region 533000, China; ^5^Department of Intensive Care Unit, The People's Hospital of Guangxi Zhuang Autonomous Region, Nanning 531000, China; ^6^Department of Surgery and Cancer, Imperial College London, Chelsea and Westminster Hospital, London SW10 9NH, UK; ^7^Department of Intensive Care Medicine, Youjiang Medical University for Nationalities, Baise, Guangxi Zhuang Autonomous Region 533000, China

## Abstract

**Background:**

LBP and fractalkine are known to be involved in the pathogenesis of ARDS. This study investigated the relationship between LBP and fractalkine in LPS-induced A549 cells and rat lung tissue in an ARDS rat model.

**Methods:**

A549 cells were transfected with LBP or LBP shRNA plasmid DNA or pretreated with SB203580 or SC-514 following LPS treatment. An ARDS rat model was established using LPS with or without LBPK95A, SB203580, or SC-514 treatment. RT-PCR, western blotting, ELISA, immunofluorescence, coimmunoprecipitation, and immunohistochemical staining were used to study the expression of fractalkine and LBP and p38 MAPK and p65 NF-*κ*B activities.

**Results:**

LPS increased LBP and reduced fractalkine. LBP overexpression further decreased LPS-induced downregulation of fractalkine and p38 MAPK and p65 NF-*κ*B activation; LBP gene silencing, SB203580, and SC-514 suppressed LPS-induced downregulation of fractalkine and p38 MAPK and p65 NF-*κ*B activation in A549 cells. LBP and fractalkine in lung tissue were increased and decreased, respectively, following LPS injection. LBPK95A, SB203580, and SC-514 ameliorated LPS-induced rat lung injury and suppressed LPS-induced downregulation of fractalkine by decreasing phospho-p38 MAPK and p65 NF-*κ*B.

**Conclusions:**

The results indicate that LBP downregulates fractalkine expression in LPS-induced A549 cells and in an ARDS rat model through activation of p38 MAPK and NF-*κ*B.

## 1. Introduction

Acute respiratory distress syndrome (ARDS) is associated with high mortality and morbidity [1, 2]. ARDS is characterized by neutrophilic inflammation, pulmonary edema, hemorrhage, alveolar-capillary destruction, and hyaline membrane formation in the lung resulting subsequently in alveolar flooding and acute respiratory failure [3, 4]. The pathogenesis of ARDS is caused by lung inflammation, which includes the accumulation of inflammatory cells, release of proteases and proinflammatory cytokines, and a sustained loss of normal alveolar capillary barrier function [5, 6]. Endotoxemia caused by Gram-negative bacterial infection is one of the causes of lung inflammation [7]. Lipopolysaccharide (LPS) is a major component of the cell wall of Gram-negative bacteria and its endotoxins [8]. Increasing lines of evidence have suggested that LPS and inflammatory cytokines are involved in the development and progression of ARDS [9, 10].

Lipopolysaccharide-binding protein (LBP), a key participant in the inflammatory response to infection, is a type I acute phase response protein that is produced by airway epithelial cells, hepatocytes, and a myriad of other cell types and enhances the recognition of endotoxin and pathogens by the immune system [11, 12]. Levels of LBP in the lung have been found to be increased in patients with ARDS [13]. LBP binds to gram-negative bacteria via the lipid A portion of the LPS which mediates its binding to the CD14 cellular receptor molecule presented by monocytes and macrophages and potentiates LPS signaling activity resulting in the production of proinflammatory cytokines that subsequently leads to lung injury [14]. However, LBP was also shown to be involved in the efficient elimination of bacteria and the loss of LBP-mediated function results in derangements of the inflammatory response and bacterial clearance mechanisms in gram-negative-induced pneumonia by delaying the influx of neutrophils [15]. This led to the conclusion that LBP might have a dual role: augmenting the inflammatory response to bacterial toxins such as LPS and contributing to bacterial elimination via associate neutrophil infiltration. The potential role of LBP in LPS-induced ARDS is not fully understood.

Fractalkine (FKN; CX3CL1) is a chemokine which functions dually as a chemoattractant and an adhesion molecule that has been linked to several types of inflammatory diseases [16, 17]. FKN, recognized as CX3C ligand (CX3CL1), is a unique chemokine of the CX3 chemokine family that acts as either a membrane bound (adhesion molecule) or a soluble (chemokine) mediator, facilitating T cell and monocyte adhesion and transmigration [18, 19]. The expression of FKN can be shown in alveolar epithelial cells, pulmonary vascular endothelial cells, fibroblasts, and airway smooth muscle cells [20–22], and it displays a role in the initiation and progression of intimate contacts of inflammatory cells with endothelium [23]. It protects the cells from Fas/FasL-mediated apoptosis in alveolar epithelial cells, inhibits inflammation-associated markers [24] such as monocyte chemoattractant protein-1 (MCP-1), and induces monocyte migration via the inhibition of stress-activated protein kinase 2/p38 and matrix metalloproteinase activities [25]. It has therefore been suggested that FKN has a novel role in inflammation. However, the anti-inflammatory effect of FKN in alveolar epithelial cells has not been documented and the role of FKN in LPS-induced ARDS is still not clear.

It is now well established that the three major mitogen-activated protein kinase (MAPK) signaling pathways, namely, p38, c-Jun NH2-terminal kinase (JNK), and extracellular signal-regulated kinase (ERK), regulate a variety of cellular activities, including proliferation, differentiation, and apoptosis, in response to different external stimuli. p38 MAPKs are stress-activated MAPKs, and both p38 MAPK and NF-*κ*B are involved in cytokine production and in the pathophysiology of ARDS [26, 27]. The p38 MAPK family members (p38, MAPK11, SAPK3, and SAPK4) are activated by inflammatory cytokines, UV radiation, and external stress such as heat shock and high osmotic stress. Once activated, the p38 MAPK pathway initiates the production of transcription factors including PAX6, p53, ATF1, ATF2, and CREB and regulates the production of proinflammatory or apoptosis-associated genes [26, 27]. Nuclear factor-*κ*B (NF-*κ*B) is a nuclear transcription factor that regulates critical cellular behavior and many cytokines by influencing biological processes of cells including inflammation, innate and adaptive immunity, and stress responses. NF-*κ*B proteins include NF-*κ*B2 p52/p100, NF-*κ*B1 p50/p105, c-Rel, RelA/p65, and RelB. Activated NF-*κ*B is translocated to the nucleus to induce target gene expression [27]. Sustained activation of NF-*κ*B is harmful by constantly generating inflammatory mediators which contribute to inflammatory diseases and inflammation-associated lung injury [28]. These signaling transduction pathways acting together create a network that activates a variety of transcription factors, which coordinately induce the transcription of many genes to affect the development of ARDS [29].

The mechanisms by which signaling pathways regulate the FKN expression to participate in the process of experimental ARDS remain unknown. Our hypothesis is that LBP is involved in LPS-induced FKN release through p38 MAPK or NF-*κ*B signaling pathway. Cultured A549 cells as an in vitro model and an LPS-induced ARDS rat as an in vivo model are used here to investigate these relevant signaling transduction pathways.

## 2. Materials and Methods

### 2.1. Cell Culture

A549 cells were obtained from the American Type Culture Collection (VR-15™). The cells were cultured in Roswell Park Memorial Institute 1640 (RPMI 1640) media (Gibco, USA), supplemented with 10% fetal bovine serum (HyClone, USA), and maintained at 37°C in a humidified atmosphere with 5% CO_2_. Cells were subcultured every 3-4 days after reaching a density of 5000 cells/cm^2^. For LPS treatment, A549 cells were seeded (5000 cells/cm^2^) into 30 mm six-well plates. Once confluent, the cells were incubated in serum-free medium for 24 h before each experiment.

### 2.2. Cell Treatment and Sample Collection

#### 2.2.1. Groupings

The cells were divided into seven groups as follows: control group (CTL); LPS group (LPS, LPS treatment at 10 *μ*g/mL, L2880, Sigma-Aldrich, USA); LPS and LBP group (LPS+LBP(+), the cells were transfected with the LBP plasmid DNA (Genechem Co., Ltd., Shanghai, China) by using Lipofectamine® 2000 transfection reagent (11668-027, Invitrogen, USA) and maintained in RPMI 1640 medium for 48 h following LPS treatment); LPS and LBP(−) group (LPS+LBP(−), the cells were transfected with the pGCU6/Neo-LBP shRNA-expressing plasmid DNA (ShLBP) which targeted the LBP mRNA sequence (5′-GCATCAGCATTTCGGTCAACC-3′) (Genechem Co., Ltd., Shanghai, China) by using Lipofectamine 2000 transfection reagent and maintained in RPMI 1640 medium for 48 h following LPS treatment); LPS and SB203580 group (LPS+SB, the cells were preincubated with 10 *μ*M SB203580 (S8307, Sigma-Aldrich, USA) for 60 minutes following LPS treatment, and the concentration of SB203580 was as described previously [30]); LPS and SC-514 group (LPS+SC, the cells were preincubated with 10 *μ*M SC-514 (SML0557, Sigma-Aldrich, USA) for 60 minutes following LPS treatment, and the concentration of SC-514 was as described previously); and tumor necrosis factor-*α* (TNF-*α*) group (TNF-*α*, TNF-*α* treatment at 50 ng/mL for positive control (H8916, Sigma-Aldrich, USA), the concentration of TNF-*α* was as described previously [31]).

The cell supernatants were collected 2 h after LPS treatment. RNA was isolated from the cells 2 hours after LPS stimulation by using an RNeasy Mini Kit (74104, Qiagen, USA). RNA integrity was checked electrophoretically and quantified by using spectrophotometry. Cell lysates were collected in cell lysis buffer (9803, Cell Signaling Technology, USA) according to the experimental conditions 1 h after LPS treatment for subsequent coimmunoprecipitation (Co-IP) and western blotting. For immunofluorescence analysis by confocal laser-scanning microscopy, the cells were fixed in 4% paraformaldehyde for 1 h after LPS treatment. The culture supernatant was harvested 24 h after LPS stimulation for the enzyme-linked immunosorbent assay (ELISA) measurement of FKN.

#### 2.2.2. Plasmid Transfection

A549 cells were allowed to grow until about 80% confluency and then transfected with LBP plasmid DNA and LBP shRNA-expressing plasmid DNA. For transfection, 4 *μ*g of plasmid and 10 *μ*L Lipofectamine 2000 were added to each well of a 6-well plate. Cells were incubated at 37°C for 48 h in a humidified incubator containing 95% air, 5% CO_2_ for 48 h, and then treated with or without 10 *μ*g/mL LPS. An empty vector was used as a vehicle control. At the same time, a group of GFP tag plasmids (including LBP plasmid DNA, LBP shRNA plasmid DNA, and empty vector) was used for parallel controls to observe the transfection efficiency under a fluorescence microscope. The cell viability was analyzed by the MTT assay to determine the cytotoxic effects of Lipofectamine 2000 transfection reagent. RT-PCR was used to detect the effect of plasmid DNA transfection on the expression of LBP gene.

### 2.3. Animal Sample Collection

The study was approved by the Committee of Animal Care and Use of Youjiang Medical University for Nationalities, and all procedures were performed according to the National Institute of Health Guidelines. The ARDS rat model was induced by intravenous injection of LPS as previously described [32]. Adult male Sprague-Dawley rats (body weight 220–250 g) were housed individually at a constant temperature (22 ± 2°C) and humidity with a 12 h light/dark cycle and free access to chow and water. A total of 50 adult male Sprague-Dawley rats were used to perform the experiments. The animals were divided into five groups randomly: control group (CTL, *n* = 10, free of neither inhibitor nor LPS treatment); LPS group (LPS, *n* = 10, induced by a tail intravenous injection of 5 mg/kg LPS); LPS and LBPK95A group (LPS+LBPK95A, *n* = 10, injected intraperitoneally with 5 mg/kg LBP inhibitory peptide LBPK95A (RVQGRWKVRASFFK, synthesized by Selleck Chemicals (Shanghai, China)) for 2 hours following 5 mg/kg LPS injection intravenously); LPS and SB203580 group (LPS+SB, *n* = 10, pretreated with 5 mg/kg SB203580 for 30 min following 5 mg/kg LPS injection intravenously); and LPS and SC514 group (LPS+SC, *n* = 10, pretreated with 5 mg/kg SC514 for 30 min following 5 mg/kg LPS injection intravenously). SB203580, SC514, and LBPK95A were used at a dose as described previously [33]. Blood samples, bronchoalveolar lavage fluid (BALF), and lung samples were collected 24 h after LPS injection. BALF was collected from the left lung by infusing PBS (4°C, 15 mL/kg) and withdrawal five times. The BALF was centrifuged at 1000 at 4°C for 15 min. After centrifugation, supernatants were immediately stored at −80°C for the determination of myeloperoxidase activity and sediments were resuspended in 50 *μ*L PBS for cell count. The upper lobe of the right lung was used for histopathological and immunohistochemical examination, the lower lobe of the right lung was used for wet-to-dry (W/D) weight calculation, and parts of right lung tissues were stored at −80°C and used to isolate total RNA and/or protein. Total RNA of the lung tissue was isolated using chloroform and the protocol of the TRIzol kit (Invitrogen, USA) according to the instructions of the manufacturer. The lung tissue was homogenized by using a sonicate homogenizer in RIPA buffer (9806, Cell Signaling Technology, USA) for western blotting. For histopathological and immunohistochemical staining, the lung was fixed in 10% neutral formaldehyde, embedded in paraffin wax, and sectioned (4 *μ*m thickness) and stained with hematoxylin and eosin (HE) or subjected to immunohistochemistry.

### 2.4. Real-Time PCR

The gene expression of FKN and glyceraldehyde-3-phosphate dehydrogenase (GAPDH) was evaluated using real-time PCR and SYBR Green I dye (Bio-Rad, Hercules, CA). Reverse transcription was performed on 2 *μ*g RNA with oligo (dT) primers in 25 *μ*L reactions by using the RevertAid First Strand cDNA Synthesis Kit (Thermo Fisher, K1622, USA) according to the manufacturer's instructions. All primers used were obtained from Invitrogen (Life Technologies, Shanghai; Table 1). After 5 min of initial activation at 95°C, PCR was carried out for 40 cycles at 95°C for 10 s and 60°C for 30s. GAPDH was performed simultaneously and used as the housekeeping gene. The threshold cycle (Ct) value was measured, and the comparative gene expression was calculated by 2^−△△Ct^ method as described previously [34].

### 2.5. ELISA

The cell supernatants, rat sera, and rat lung tissue homogenates were harvested and stored at −80°C. The levels of FKN were measured by the human CX3CL1/fractalkine Quantikine ELISA kit (DCX310, R&D Systems Inc., Minneapolis, USA) and rat fractalkine (CX3CL1) ELISA kit (ab100761, Abcam Ltd., Cambridge, United Kingdom) according to the manufacturer's instructions. The levels of LBP were measured by the human LBP DuoSet ELISA kit (DY008, R&D Systems Inc., Minneapolis, USA) and rat LBP ELISA kit (CSB-E11184r, Cusabio, Wuhan, China) according to the manufacturer's instructions. The BALF supernatants and rat lung tissue homogenates were harvested and stored at −80°C. The levels of myeloperoxidase activity were measured by the rat MPO ELISA kit (HK105-01, Hycult Biotech, USA) according to the manufacturer's instructions.

### 2.6. Western Blotting

Extracts containing equal amounts of total protein (20 *μ*g) were resolved by 10% SDS-PAGE and transferred to nitrocellulose membranes. After blocking with 5% nonfat milk solution for 60 min, membranes were incubated with specific antibodies overnight at 4°C. The antibodies used were against the following antigens: LBP (anti-human LBP antibody; 1 : 1000 dilution, ab169776, Abcam Ltd., Cambridge, United Kingdom), LBP (anti-rat LBP antibody; 1 : 1000 dilution, sc-14666, Santa Cruz Biotechnology), p38 MAPK (1 : 2000 dilution, 8690, Cell Signaling Technology), phospho-p38 MAPK (1 : 2000 dilution, 9910, Cell Signaling Technology), phospho-p65 (1 : 2000 dilution, 9936, Cell Signaling Technology), FKN (1 : 1000 dilution, ab25088, Cell Signaling Technology), and beta-actin (1 : 2000 dilution, SC-47778, Santa Cruz Biotechnology). After primary antibody incubation, the membranes were incubated with horseradish peroxidase conjugation (9936, Cell Signaling Technology). Detection was accomplished by using an enhanced chemiluminescence detection system. The images of western blots were scanned by Quantity One software, and the original intensity of each specific band was quantified with freeware image analysis software, NIH Image (National Institute of Health, Bethesda MD, USA).

### 2.7. Immunofluorescence Analysis by Confocal Laser-Scanning Microscopy (CLSM)

A549 cells were fixed in 4% paraformaldehyde for 30 min at room temperature, permeabilized by 0.1% Triton X-100 in PBS, blocked with 4% bovine serum albumin (BSA), and incubated with primary antibody (dilution ratios: phospho-p38 MAPK—1 : 500, phospho-p65—1 : 500, p38 MAPK—1 : 1000 dilution, p65—1 : 1000, and FKN—1 : 1000) overnight at 4°C. Cells were washed and incubated with Alexa Fluor® 594-conjugated goat anti-mouse/488-conjugated goat anti-rabbit secondary antibodies (Jackson ImmunoResearch Laboratories Inc., West Grove, PA, USA) for 50 min in the dark. Cells were washed three times, mounted with propidium iodide- (PI-) containing mounting media (ZSGB-BIO, Beijing, China), and visualized using a confocal laser scanning microscope. FKN-positive cell numbers were counted in every 300 cells, and then the FKN-positive cell ratio was calculated.

### 2.8. Coimmunoprecipitation (Co-IP)

A Universal Magnetic Co-IP Kit (54002, Active Motif, Carlsbad, CA, USA) was used for Co-IP experiments. Three micrograms of LBP antibody (ab169776, Abcam Ltd., Cambridge, United Kingdom) was added to 500 *μ*g of extracted proteins. The mixture was incubated at 4°C for 60 min with gentle mixing. Then, 25 *μ*L of Magnetic Protein G Beads was added and incubated at 4°C overnight. The mixture was centrifuged at 4000 rpm for 30 seconds at 4°C. The supernatant was discarded, and the Co-IP products were washed four times with PBS. After the final wash, the precipitates were resuspended in 20 *μ*L of sample buffer for western blotting assay. Three micrograms of rabbit IgG (A7016, Beyotime Biotechnology, Nantong, China) and no antibody were used instead of anti-LBP antibody for negative control and blank control, respectively.

### 2.9. Arterial Blood Gas Analysis

Gas exchange was assessed by measuring arterial pressure of oxygen (PaO_2_), with an anti-STAT 300 portable analyzer (Abbott, USA), and PaO_2_/FiO_2_ ratios were calculated.

### 2.10. Wet-to-Dry Weight Ratio

After the animals were euthanized at 24 h, the chest cavity was opened and the lower lobe of the right lung was ligated and excised. The lung specimen was then rinsed briefly in phosphate-buffered saline (PBS), blotted, and weighed to determine the “wet” weight. Subsequently, the lungs were dried in an oven at 80°C for 48 h to obtain the “dry” weight. The ratio of wet-to-dry (W/D) weight was then calculated.

### 2.11. Neutrophil Number

The total cell numbers and neutrophil levels in the BALF were counted with a cell counter. The ratio of neutrophils/total cells was calculated.

### 2.12. Immunohistochemistry

Immunohistochemical staining for FKN expression was carried out by using 4 *μ*m thick paraffin-embedded sections of lung tissue. The lung tissue were mounted on charged slides, fixed in 10% neutral formaldehyde, and immersed in 3% (*v*/*v*) hydrogen peroxide in PBS for 10 min to prevent endogenous peroxidase activity. The antigens were activated by microwaving (the slides were placed in the boiling citrate antigen retrieval solution (pH 6.0) and then were heated for ten minutes and subsequently cooled to room temperature) and incubated overnight with a polyclonal FKN antibody (1 : 250, ab25088, Abcam Ltd., Cambridge, United Kingdom) at 4°C. The slides were further incubated for 30 min with a secondary HRP-En Vision IgG antibody (Boster Biotech, China) for 30 min at room temperature. Specific staining was detected by the two-step streptavidin-peroxidase method (Non-Biotin HRP Direction System). FKN was visualized by incubating the sections with a solution of 3,3′-diaminobenzidine for 10 min. Negative controls were incubated with normal rabbit serum instead of the primary antibody. Then counterstaining was performed with hematoxylin. The immunoreactive cells were counted in at least eight fields and expressed as a positive cell ratio to the alveolar epithelium.

### 2.13. Statistical Analysis

Values were expressed as means ± standard errors of the means. Statistical analysis was performed using SPSS for windows (version 16.0, Chicago, USA). Differences between the 2 groups were compared by Student's *t*-test. When multiple groups were compared, analysis of variance was used and, if significance was observed, post hoc comparisons between the groups using the method of Games-Howell were performed. *p* value < 0.05 was considered significant.

## 3. Results

### 3.1. LBP Gene Overexpression Further Decreased LPS-Induced Downregulation of FKN mRNA and Protein Expression; LBP Gene Silencing, SB203580, and SC-514 Suppressed LPS-Induced Downregulation of FKN mRNA and Protein Expression, Respectively, in A549 Cells

**Figure 1:**
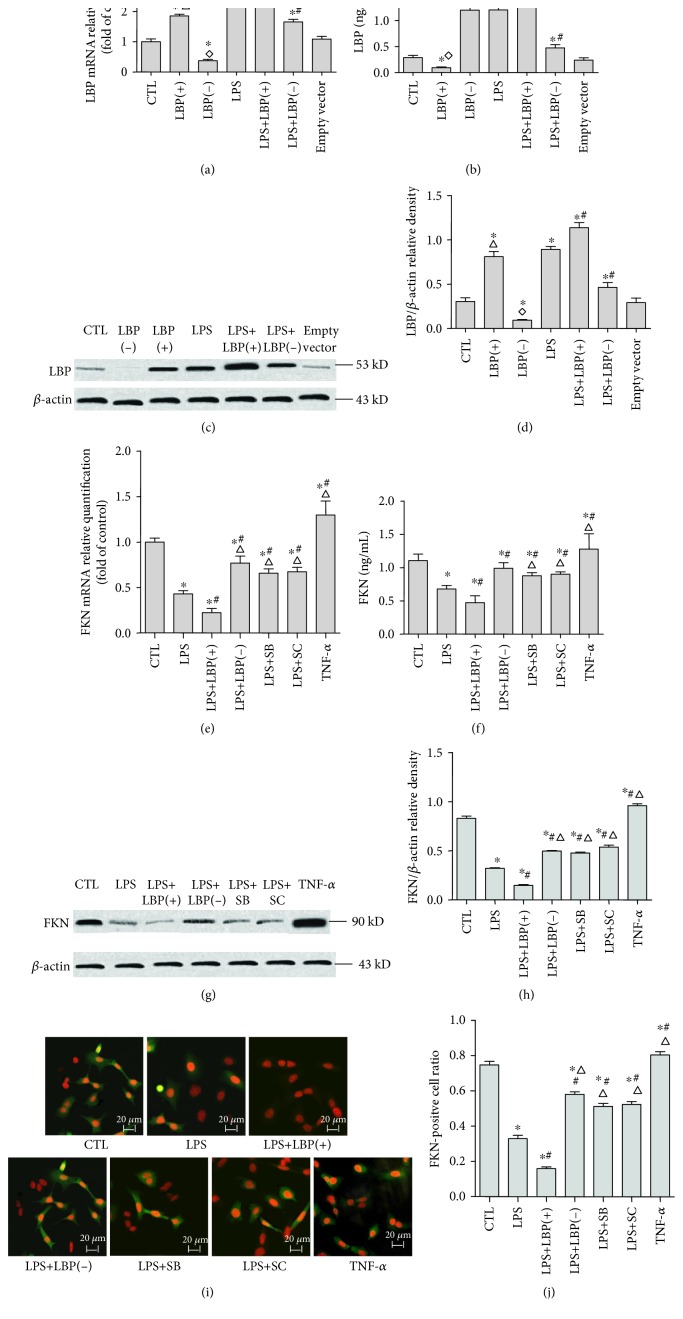
LBP gene overexpression further decreased LPS-induced downregulation of FKN mRNA and protein expression; LBP gene silencing, SB203580, and SC-514 suppressed LPS-induced downregulation of FKN mRNA and protein expression, respectively, in A549 cells. RT-PCR and ELISA were used to analyze the LBP and FKN mRNA and protein expression, respectively, in A549 cells. The LBP mRNA expression is shown in (a). The relative LBP mRNA levels (normalized to GAPDH mRNA) in LPS-stimulated cells are significantly higher than those in control cells (*p* < 0.05). The LBP mRNA levels of the LBP(+) group and LBP(−) group are upregulated and downregulated, respectively, when compared with the control group (*p* < 0.05 in all cases). The LBP mRNA levels of the LPS+LBP(+) group of cells are upregulated compared with those of the control group and LPS group (*p* < 0.05 in all cases). The LBP mRNA levels of the LPS+LBP(−) group of cells are upregulated compared with those of the control group and downregulated compared with those of the LPS group (*p* < 0.05 in all cases). The LBP mRNA level of the empty vector group is not different from that of the control group (*p* > 0.05). The FKN mRNA expression is shown in (e). The relative FKN mRNA levels (normalized to GAPDH mRNA) in LPS-stimulated cells are significantly lower than those in control cells (*p* < 0.05). The FKN mRNA levels of the LPS+LBP(+) group of cells are downregulated compared with those of the control group and the LPS group (*p* < 0.05 in all cases). The FKN mRNA levels of the LPS+LBP(−), LPS+SB, and LPS+SC groups of cells are downregulated compared with those of the control group and upregulated compared with those of the LPS group (*p* < 0.05 in all cases). The FKN mRNA levels of the TNF-*α* group are upregulated compared with those of the control group (*p* < 0.05). The results of ELISA and western blotting in (b), (c), and (d) demonstrate that the LPS treatment significantly increases the levels of LBP protein expression (*p* < 0.05). The LBP protein levels of the LBP(+) group and LBP(−) group are upregulated and downregulated, respectively, compared with those of the control group (*p* < 0.05 in all cases). The LBP protein levels of the LPS+LBP group of cells are upregulated compared with those of the control group and the LPS group (*p* < 0.05 in all cases). The LBP protein levels of the LPS+LBP(−) group of cells are upregulated compared with those of the control group and downregulated compared with those of the LPS group (*p* < 0.05 in all cases). ELISA and western blotting assay in (f), (g), and (h) demonstrate that the LPS treatment significantly reduces the levels of FKN protein expression (*p* < 0.05). The FKN protein levels of the LPS+LBP group of cells are downregulated compared with those of the control group and the LPS group (*p* < 0.05 in all cases). The FKN protein levels of the LPS+LBP(−), LPS+SB, and LPS+SC groups of cells are downregulated compared with those of the control group and upregulated compared with those of the LPS group (*p* < 0.05 in all cases). The FKN protein levels of the TNF-*α* group are higher than those of the control group (*p* < 0.05). Immunofluorescence staining results in (i) and (j) confirmed that TNF-*α* induced a high FKN-positive cell ratio in A549 cells. LPS decreased FKN-positive cell ratio in A549 cells; LBP gene overexpression further decreased FKN-positive cell ratio which was inhibited by LPS, LBP gene silencing, and SB203580. Also, SC-514 reduced the inhibition of LPS (*p* < 0.05 in all cases). The photomicrographs are 200x magnification. Data are presented as means ± SD of six independent experiments (*n* = 6). ∗ represents *p* < 0.05 when compared with that in the CTL; # represents *p* < 0.05 when compared with that in the LPS; △ represents *p* < 0.05 when compared with that in the LPS+LBP(+). ^∗^*p* < 0.05 when compared with that in the control group; ◇ represents *p* < 0.05 when compared with that in the LPS+LBP(−) group; △ represents *p* < 0.05 when compared with that in the LPS+LBP(+).

RT-PCR, western blotting, and ELISA were used to analyze the LBP and FKN mRNA and protein expression, respectively, in A549 cells. The LBP mRNA expression is shown in Figure 1(a). The relative LBP mRNA levels (normalized to GAPDH mRNA) in LPS-stimulated cells were 1.25 ± 0.18 times higher than those in control cells (*p* < 0.05, *n* = 6). The LBP mRNA levels of the LBP(+) group are 1.46 ± 0.27 times higher than those of the control group (*p* < 0.05, *n* = 6).The LBP mRNA levels of the LBP(−) group were decreased by 62.67 ± 15.55% compared with those of the control group (*p* < 0.05, *n* = 6). The LBP mRNA levels of the LPS+LBP(+) group of cells were 2.96 ± 0.79 and 1.30 ± 0.28 times higher than those of the control group and the LPS group, respectively (*p* < 0.05, *n* = 6). The LBP mRNA levels of the LPS+LBP(−) group of cells were 2.65 ± 0.21 times higher and were decreased by 70.22 ± 11.25% compared with those of the control group and the LPS group, respectively (*p* < 0.05, *n* = 6, in all cases; Figure 1(a)). There is no difference between the empty vector group and the control group (*p* > 0.05, *n* = 6). The FKN mRNA expression is shown in Figure 1(e). The relative FKN mRNA levels (normalized to GAPDH mRNA) in LPS-stimulated cells decreased by 56.87 ± 16.42% compared with those seen in control cells (*p* < 0.05). The FKN mRNA levels of the LPS+LBP(+) group of cells decreased by 77.52 ± 9.05% and 20.65 ± 15.10% compared with those of the control group and the LPS group, respectively (*p* < 0.05, *n* = 6). The FKN mRNA levels of the LPS+LBP(−), LPS+SB, and LPS+SC groups of cells decreased by 23.09 ± 22.07%, 34.22 ± 21.53%, and 32.75 ± 20.86% compared with those of the control group and increased by 33.78 ± 19.80%, 22.65 ± 7.57%, and 24.12 ± 10.58% compared with those of the LPS group, respectively (*p* < 0.05, *n* = 6). The FKN mRNA levels of the TNF-*α* group increase by 30.07 ± 4.36% compared with those of the control group (*p* < 0.05, *n* = 6). The results of western blotting and ELISA in Figures 1(b), 1(c), and 1(d) demonstrate that after the LPS treatment, LBP plasmid DNA transfection increased 2.01 ± 1.33 and 2.20 ± 1.01 times of LBP protein expression, respectively, in cells (*p* < 0.05) and increased 3.58 ± 1.45 and 3.56 ± 1.45 times of LBP protein expression, respectively, in supernatants (*p* < 0.05). The LBP protein levels of the LBP(−) group were downregulated by 66.44 ± 15.04% and 61.44 ± 21.21% compared with those of the control group in cells and supernatants, respectively (*p* < 0.05). The LBP protein levels of the LPS+LBP(+) group of cells were upregulated by 28.85 ± 23.11% and 48.22 ± 19.2% compared with those of the LPS group in cells and supernatants, respectively (*p* < 0.05). The LBP protein levels of the LPS+LBP(−) group of cells were downregulated by 46.88 ± 19.63% and 60.70 ± 11.37% in cells and supernatants, respectively, compared with those of the LPS group (*p* < 0.05, *n* = 6). Western blotting and ELISA assay results demonstrate that TNF-*α* increased by 15.18 ± 22.36% and 15.05 ± 12.28% of FKN protein expression in cells and supernatants, respectively; however, the LPS treatment significantly reduced by 65.48 ± 8.45% and 38.25 ± 7.38% of FKN protein expression in cells and supernatants, respectively (*p* < 0.05, *n* = 6). Compared with the control group and the LPS group, the FKN protein levels of the LPS+LBP group of cells were downregulated by 87.28 ± 6.32% and 64.36 ± 12.06% in cells and 10.24 ± 6.52% and 47.37 ± 23.0% in supernatants (*p* < 0.05, *n* = 6). The FKN protein levels of the LPS+LBP(−), LPS+SB, and LPS+SC groups of cells were downregulated by 37.66 ± 14.55%, 40.37 ± 18.34%, and 31.77 ± 10.97%, respectively, in cells and downregulated by 57.47 ± 7.54%, 20.05 ± 8.25%, and 18.01 ± 5.92%, respectively, in supernatants compared with those of the control group. The FKN protein levels of the LPS+LBP(−), LPS+SB, and LPS+SC groups of cells were upregulated by 87.20 ± 52.05%, 84.43 ± 77.470%, and 102.2 ± 25.80%, respectively, in cells and upregulated by 30.28 ± 14.69%, 30.03 ± 10.57%, and 33.74 ± 12.5%, respectively, in supernatants compared with those of the LPS group (*p* < 0.05, *n* = 6, in all cases). FKN positive cell ratios were calculated to further verify the expression of FKN protein in the cells of each group. In order to be a positive control, TNF-*α* induced a high FKN-positive cell ratio in A549 cells. LPS decreased 55.49 ± 5.48% of the FKN-positive cell ratio in A549 cells. LBP gene overexpression further decreased by 54.36 ± 21.25% of the FKN-positive cell ratio that was able to be inhibited by LPS. In addition, LBP gene silencing, SB203580, and SC-514 reduced by 84.36 ± 27.77%, 54.84 ± 28.58%, and 58.48 ± 44.65% of the inhibition of LPS, respectively, by the different groups (*p* < 0.05, *n* = 6). Both FKN mRNA and protein expression were downregulated following LPS treatment with or without pretreatment of cultured A549 cells with LBP gene overexpression or silencing, SB203580, and SC-514 when compared with control cells.

### 3.2. LBP Gene Overexpression Further Increased LPS-Induced p38 MAPK and p65 NF-*κ*B Activation; LBP Gene Silencing, SB203580, and SC-514 Suppressed LPS-Induced p38 MAPK and p65 NF-*κ*B Activation, Respectively, in A549 Cells

**Figure 2:**
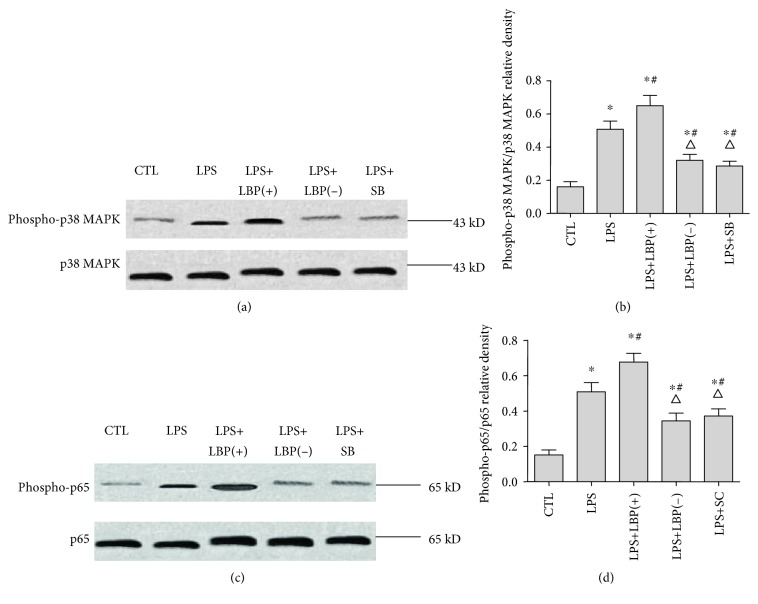
The expressions of phospho-p38 MAPK and phospho-p65 in A549 cells with and without plasmid transfection or inhibitors following LPS treatment. Western blotting was used to image the expression of phospho-p38 MAPK and phospho-p65 in A549 cells with and without plasmid transfection or inhibitors following LPS treatment. A549 cells of the LPS+LBP and LPS+LBP(−) groups were transfected with LBP plasmid DNA or LBPshRNA-expressing plasmid DNA, respectively, for 48mins following LPS treatment. A549 cells of the LPS+SB and LPS+SC groups were pretreated with SB203580 or SC-514 for 60 mins following LPS treatment. The cells were collected with cell lysis buffer 1 h after LPS stimulation for western blotting. The figure shows that there is increased expression of phospho-p38 MAPK and phospho-p65 in A549 following LPS treatment. Transfection with LBP plasmid DNA increased the expression of phospho-p38 MAPK and phospho-p65. Transfection with LBP shRNA plasmid DNA decreased the expression of phospho-p38 MAPK and phospho-p65. Pretreatment with SB203580 reduced the expression of phospho-p38 MAPK, and pretreatment with SC-514 reduced the expression of phospho-p65 protein. The relative densities of phospho-p38 MAPK/*β*-actin (a and c) and phospho-p65/*β*-actin (b and d). ∗ represents *p* < 0.05 when compared with that in the control group; # represents *p* < 0.05 when compared with that in the LPS group; △ represents *p* < 0.05 when compared with that in the LPS+LBP(+). Data are presented as means ± SD of six independent experiments (*n* = 6).

Western blotting was used to image the expression of phospho-p38 MAPK and phospho-p65 in A549 cells with and without plasmid transfection or inhibitors following LPS treatment. The results show that there were increases of 2.52 ± 0.86 and 2.73 ± 1.01 times of the expression of phospho-p38 MAPK and phospho-p65, respectively, in A549 following LPS treatment (*p* < 0.05, *n* = 6, in all cases). Transfection with LBP plasmid DNA increased the expression by 29.31 ± 13.83% and 38.73 ± 23.47% of phospho-p38 MAPK and phospho-p65, respectively (*p* < 0.05, *n* = 6, in all cases). Transfection with LBP shRNA plasmid DNA decreased the expression of phospho-p38 MAPK by 37.20 ± 7.60% and the expression of phospho-p65 by 30.14 ± 25.94% (*p* < 0.05, *n* = 6). Pretreatment with SB203580 reduced the expression of phospho-p38 MAPK by 43.58 ± 7.90%, and pretreatment with SC-514 reduced the expression of phospho-p65 protein by 24.03 ± 21.82% (*p* < 0.05, *n* = 6) (Figure 2).

### 3.3. LBP Gene Overexpression Promoted Phospho-p38 MAPK and Phospho-p65 Translocation, LBP Gene Silencing Inhibited Both Phospho-p38 MAPK and Phospho-p65 Translocation, and SB203580 and SC-514 Inhibited Phospho-p38 MAPK and Phospho-p65 Translocation, Respectively, in Response to LPS Stimulation

**Figure 3:**
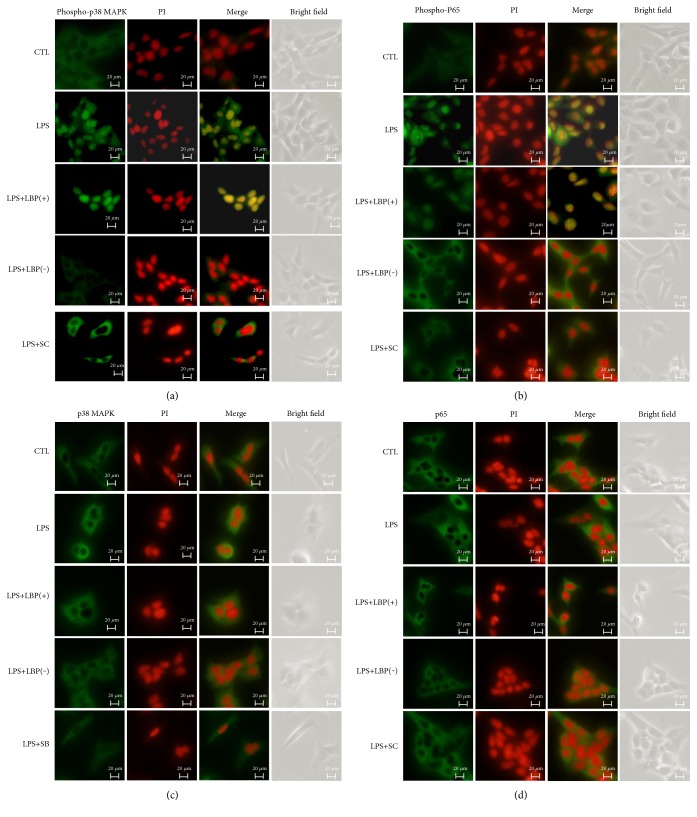
(a) LBP promoted phospho-p38 MAPK translocation, LBP shRNA, and SB203580 inhibited phospho-p38 MAPK translocation in response to LPS stimulation, and (b) LBP promoted phospho-p65 translocation, LBP shRNA, and SC-514 inhibited phospho-p65 translocation in response to LPS stimulation. Immunohistochemical staining was used to image the effect of LBP overexpression, LBP silencing, SB203580, and SC-514 on the activation of p38 MAPK and NF-B in A549 cells. The cells were fixed in 4% paraformaldehyde and permeabilized. Following incubation with anti-phospho-p38 MAPK or anti-phospho-p65 antibody overnight at 4°C, a fluorescent secondary antibody was used for nuclear staining. The cell slides were mounted with propidium iodide- (PI-) containing mounting media and visualized using a confocal laser scanning microscope. The photomicrographs are at 400x magnification.

Confocal imaging revealed that p38 MAPK, p65, phospho-p38 MAPK, and phospho-p65 were detectable within A549 cells. LPS induced the translocation of activated p38 MAPK (phospho-p38 MAPK) and p65 (phospho-p65) into the cell nuclei. Transfection with the LBP plasmid DNA promoted p38 and p65 translocation. Transfection with the LBP shRNA plasmid DNA inhibited p38 and p65 translocation. Preincubation of the cells with SB203580 inhibited p38 translocation, and SC-514 inhibited the nuclear translocation of p-65 (Figure 3).

### 3.4. LBP Gene Overexpression Enhanced the Interaction between LBP and Phospho-p38 MAPK and LBP and Phospho-p65; LBP Gene Silencing Inhibited the Interaction between LBP and Phospho-p38 MAPK and LBP and Phospho-p65; SB203580 and SC-514 Inhibited the Interaction between LBP and Phospho-p38 MAPK and LBP and Phospho-p65, Respectively, in A549 Cells

**Figure 4:**
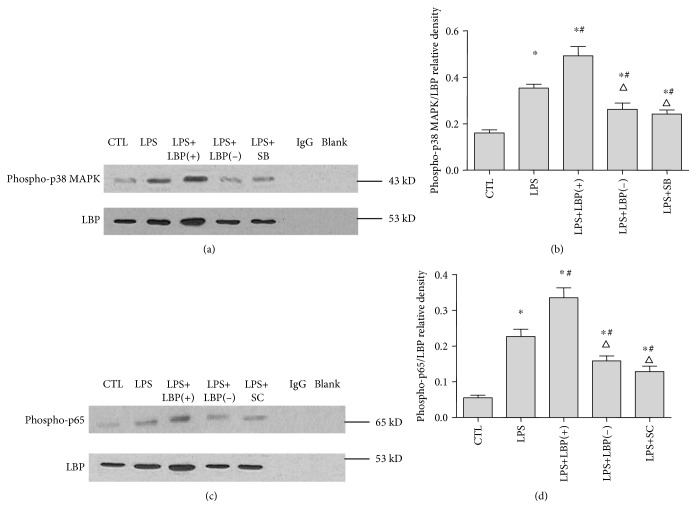
LBP gene overexpression enhanced the interaction between LBP and phospho-p38 MAPK and LBP and phospho-p65. LBP gene silencing inhibited the interaction between LBP and phospho-p38 MAPK and LBP and phospho-p65. SB203580 and SC-514 inhibited the interaction between LBP and phospho-p38 MAPK and LBP and phospho-p65, respectively, in A549 cells. Coimmunoprecipitation and western blotting were used to detect the interaction between LBP and phospho-p38 MAPK and LBP and phospho-p65. Phospho-p38 MAPK/LBP and phospho-p65/LBP relative densities were calculated to measure the strength of protein interactions. The result shows that LBP interacted with phospho-p38 MAPK and phospho-p65 weakly in the control group of cells (*p* < 0.05 in all cases). The interaction effects were strengthened by LPS stimulation. Transfection with LBP plasmid DNA and LBP shRNA-expressing plasmid DNA, respectively, strengthen and weaken the stimulatory effect of LPS (*p* < 0.05 in all cases). SB203580 inhibited the interaction between LBP and phospho-p38 MAPK; SC-514 also inhibited the interaction between LBP and phospho-p65 (*p* < 0.05 in all cases). ∗ represents *p* < 0.05 when compared with that in the control group; # represents *p* < 0.05 when compared with that in the LPS group; △ represents *p* < 0.05 when compared with that in the LPS+LBP(+). Data are presented as means ± SD of six independent experiments (*n* = 6).

Coimmunoprecipitation and western blotting were used to detect the interaction between LBP and phospho-p38 MAPK and LBP and phospho-p65. Phospho-p38 MAPK/LBP and phospho-p65/LBP relative densities were calculated to measure the strength of protein interactions. The result shows that LBP interacted with phospho-p38 MAPK and phospho-p65 weakly in the control group of cells. The interaction effects between LBP and phospho-p38 MAPK and LBP and phospho-p65 were strengthened 1.26 ± 0.36 and 3.29 ± 0.66 times, respectively, by LPS stimulation (*p* < 0.05, *n* = 6, in all cases). Transfection LBP plasmid DNA and LBP shRNA-expressing plasmid DNA strengthened by 38.89 ± 18.75% (phospho-p38 MAPK) and 50.49 ± 24.38% (phospho-p65) and weakened by 26.63 ± 12.05% (phospho-p38 MAPK) and 29.95 ± 3.65% (phospho-p65) of the stimulation effect of LPS, respectively (*p* < 0.05, *n* = 6, in all cases). SB203580 inhibited the interaction between LBP and phospho-p38 MAPK by 31.78 ± 7.51%; SC-514 also inhibited the interaction between LBP and phospho-p65 by 43.62 ± 8.35% (*p* < 0.05, *n* = 6, in all cases). There was no phospho-p38 MAPK or phospho-p65 and LBP expression in the IgG and blank groups (Figure 4).

### 3.5. LBPK95A, SB203580, and SC-514 Suppressed LPS-Induced Downregulation of FKN Gene Expression, Respectively, in the ARDS Rat Model

**Figure 5:**
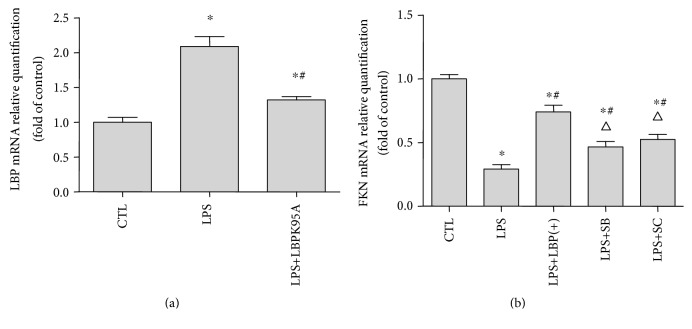
Expression of LBP and FKN mRNA in ARDS rat lung homogenates. The lung tissue was homogenized by sonicating in RIPA buffer. Total RNA of lung homogenates was isolated using chloroform, and the protocol of TRIzol kit was used according to the manufacturer's instructions. RT-PCR was used to analyze the LBP and FKN mRNA expression in ARDS rat lung tissues. The results suggest that LBPK95A, SB203580, and SC-514 suppressed the LPS-induced FKN reduction. ∗ represents *p* < 0.05 when compared with that in the CTL group rats; # represents *p* < 0.05 when compared with that in the LPS group rats; △ represents *p* < 0.05 when compared with that in the LPS+LBPK95A rats (*n* = 10).

RT-PCR was used to analyze the LBP and FKN mRNA expression in the lung tissues of the ARDS rat model. The relative LBP mRNA levels of the LPS and LPS+LBPK95A groups were 2.09 ± 0.34 and 1.322 ± 0.12-fold higher, respectively, when compared to those of the control group (*p* < 0.05, *n* = 10, in all cases). The relative LBP mRNA level was inhibited by 77.10 ± 41.52% by LBPK95A compared with that of the LPS group (*p* < 0.05, *n* = 10) (Figure 5(a)). The relative FKN mRNA levels observed in LPS-induced rats were decreased by 70.72 ± 12.34% than those in the control group (*p* < 0.05, *n* = 10). The FKN mRNA levels of the lung tissue in the LPS+LBPK95A, LPS+SB, and LPS+SC groups of rat were downregulated by 25.90 ± 19.98%, 53.47 ± 19.05%, and 47.387 ± 13.69%, respectively, compared with those in the control group and upregulated by 44.82 ± 8.20%, 17.25 ± 3.32%, and 23.34 ± 12.01%, respectively, compared with those in the LPS group (*p* < 0.05, *n* = 10, in all cases) (Figure 5(b)).

### 3.6. LBPK95A, SB203580, and SC-514 Ameliorated LPS-Induced Lung Injury and Inflammation in the ARDS Rat Model

**Figure 6:**
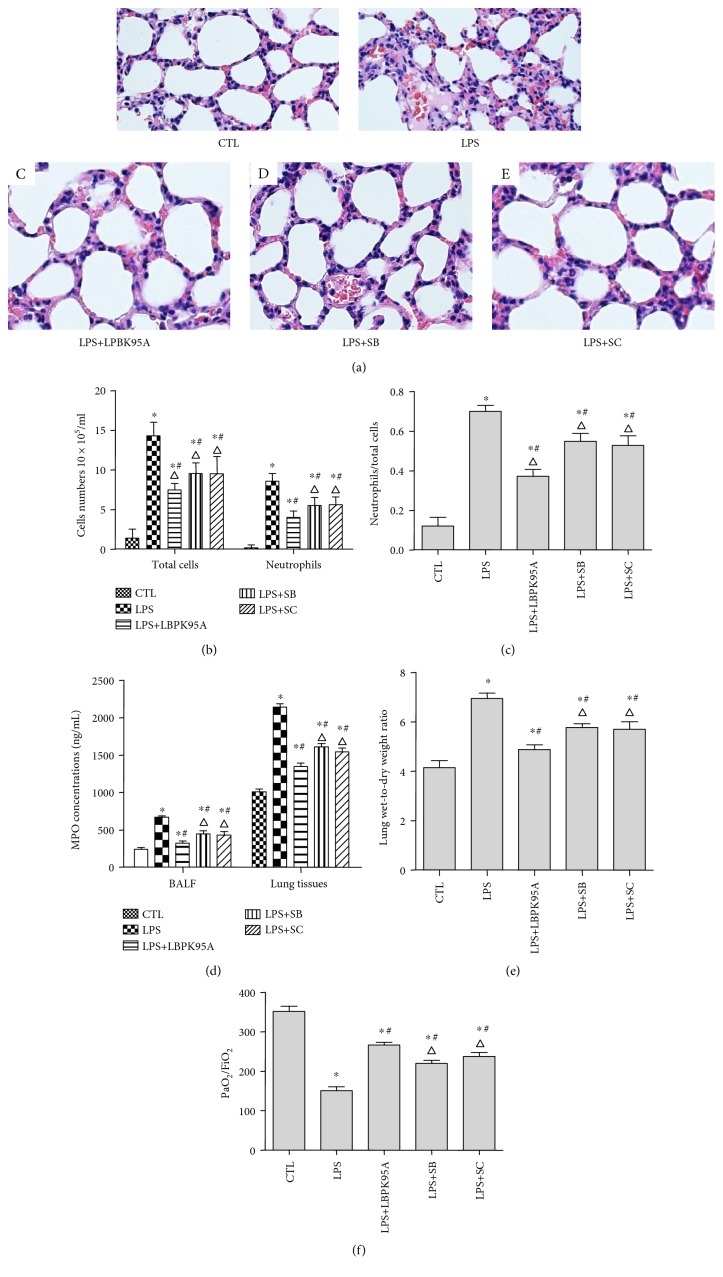
SB203580 and SC-514 ameliorated LPS-induced lung injury and inflammation. Hematoxylin and eosin staining was used to determine the LPS-induced lung injury and inflammatory response in the lung tissue of the ARDS rat model. (a) The control group rats (A) had a distinct framework with complete alveolar walls and interstitium without exfiltration. The LPS group rats (B) showed edema, neutrophil infiltration, hemorrhage, moderate bronchiole epithelial desquamation, and minimal hyaline membrane formation. Histotological analysis also show that rats receiving LBPK95A, SB203508, or SC514 injection had substantially less inflammatory cell infiltration, edema, hemorrhage, and thickness of alveolar walls in comparison to rats treated with LPS ((C), (D), and (E), resp.). The photomicrographs are at 400x magnification. ((b) and (c)) The total number of cells, neutrophil number, and neutrophil ratio in BALF were higher in the LPS group than that in the control group and lower in the LPS+LBPK95A, LPS+SB, and LPS+SC groups than that in the LPS group (*p* < 0.05, *n* = 10, in all cases). The MPO of lung tissues and BALF was determined by ELISA. The results show that the level of MPO concentration in the LPS group was significantly higher than that in the control group (*p* < 0.05). (d) However, the MPO concentration level in the LPS+LBPK95A, LPS+SB, and LPS+SC groups was significantly lower than that in the LPS group (*p* < 0.05 in all cases). (e) The LPS group had a significantly higher W/D ratio than the control group, and the W/D ratio in the LPS+LBPK95A, LPS+SB, and LPS+SC groups were significantly lower than that in the LPS group (*p* < 0.05 in all cases). The LPS group had a lower PaO_2_/FiO_2_ ratio than the control group. (f) The LPS+LBPK95A, LPS+SB, and LPS+SC groups had higher PaO_2_/FiO_2_ ratios than the LPS group (*p* < 0.05 in all cases). ∗ represents *p* < 0.05 when compared with that in the CTL group of rats; # represents *p* < 0.05 when compared with that in the LPS group of rats; △ represents *p* < 0.05 when compared with that in the LPS+LBPK95A group of rats (*n* = 10).

Pathological tissue sections stained by HE showed that LPS induced rat lung tissue injury and inflammation as observed by the increased tissue edema, neutrophil infiltration, and hemorrhage. The inhibitors, LBPK95A, SB203580, and SC-514, were able to reduce the lung injury and inflammatory response observed (Figure 6(a)). To assess the effect of LPS, LBPK95A, SB203580, and SC-514 on lung inflammation, BALF was collected from the rats in each group, the total number of cells, and the number of neutrophils and those that were activated was counted. As illustrated in Figure 6(b), the total number of cells in BALF was markedly higher in the LPS group than that in the control group (control group: 1.39 ± 1.14 × 10^5^/mL, LPS group: 14.33 ± 1.70 × 10^5^/mL; *p* < 0.001, *n* = 10, in all cases). The total number of cells in BALF decreased by 48.76 ± 3.29%, 33.20 ± 5.09%, and 32.19 ± 9.11% in the LPS+LBPK95A, LPS+SB, and LPS+SC groups when compared to that in the LPS group, respectively (*n* = 10 in all cases). To further evaluate the effect of acute inflammation, a neutrophil count was performed. As expected, there was a marked increase in all the inflammation-related cells in BALF after LPS injection. The number of neutrophils was higher in the LPS group than that in the control group (control group: 0.18 ± 0.004 × 10^5^/mL, LPS group: 8.6 ± 0.9 × 10^5^/mL; *p* < 0.05, *n* = 10). The number of neutrophils decreased by 53.38 ± 5.49%, 36.10 ± 7.65%, and 34.82 ± 5.54% in the LPS+LBPK95A, LPS+SB, and LPS+SC groups when compared to that in the LPS group, respectively (*p* < 0.001, *n* = 10, in all cases). The number of neutrophils was also 37.14 ± 6.43% and 41.38 ± 18.54% higher in the LPS+SB and LPS+SC groups than that in the LPS+LBPK95A group, respectively (*p* < 0.001, *n* = 10 in all cases). The changes of neutrophil ratio were consistent with the changes in the total cell number and neutrophil number.

The neutrophil ratio was higher in the LPS group than that in the control group (control group: 0.12 ± 0.13, LPS group: 0.70 ± 0.09; *p* < 0.001, *n* = 10). The neutrophil ratio decreased by 32.73 ± 11.81, 15.12 ± 10.18, and 17.20 ± 12.98% in the LPS+LBPK95A, LPS+SB, and LPS+SC groups when compared to that in the LPS group, respectively (*p* < 0.001, *n* = 10, in all cases) (Figure 6(c)). Activated neutrophils were implicated in the pathophysiology of lung injury and myeloperoxidase (MPO) which is an indicator of neutrophil accumulation [35]. The MPO levels in lung tissues and BALF were determined by ELISA. The results show that the level of MPO concentration in the LPS group increased 2.05 ± 0.90 times in lung tissues and 1.13 ± 0.13 times in BALF than that in the control group (*p* < 0.001, *n* = 10). However, the MPO activity level in the LPS+LBPK95A, LPS+SB, and LPS+SC groups was decreased by 52.43 ± 8.05, 34.42 ± 15.35, and 36.90 ± 16.42%, respectively, in lung tissues and 36.96 ± 4.09, 24.80 ± 4.11, and 27.59 ± 8.32%, respectively, in BALF than that in the LPS group (*p* < 0.001, *n* = 10, in all cases), indicating that LBPK95A, SB, and SC inhibited MPO activity induced by LPS, respectively (Figure 6(d)).

The W/D ratio is an indicator of pulmonary edema [36]. The W/D ratio of the LPS group was 73.56 ± 34.71% higher than that of the control group, indicating the presence of pulmonary edema (*p* < 0.05, *n* = 10). However, the W/D ratio in the LPS+LBPK95A, LPS+SB, and LPS+SC groups was 29.44 ± 9.47, 16.16 ± 10.99, and 17.33 ± 16.00% lower than that in the LPS group, respectively, indicating that LBPK95A, SB, and SC attenuated the degree of pulmonary edema induced by LPS (*p* < 0.05, *n* = 10, in all cases) (Figure 6(e)).

The PaO_2_/FiO_2_ ratio is an important index for the diagnosis of ARDS. The PaO_2_/FiO_2_ ratio of the LPS group was 57.48 ± 4.57% lower than that of the control group (*p* < 0.05, *n* = 10). However, the PaO_2_/FiO_2_ ratio of the LPS+LBPK95A, LPS+SB, and LPS+SC groups was 81.33 ± 27.06, 9.16 ± 22.35, and 9.95 ± 16.01% higher than that of the LPS group, respectively, *p* < 0.05, *n* = 10, in all cases) (Figure 6(f)).

### 3.7. LBPK95A, SB203580, and SC-514 Suppressed LPS-Induced Downregulation of FKN Expression, Respectively, in the ARDS Rat Model

**Figure 7:**
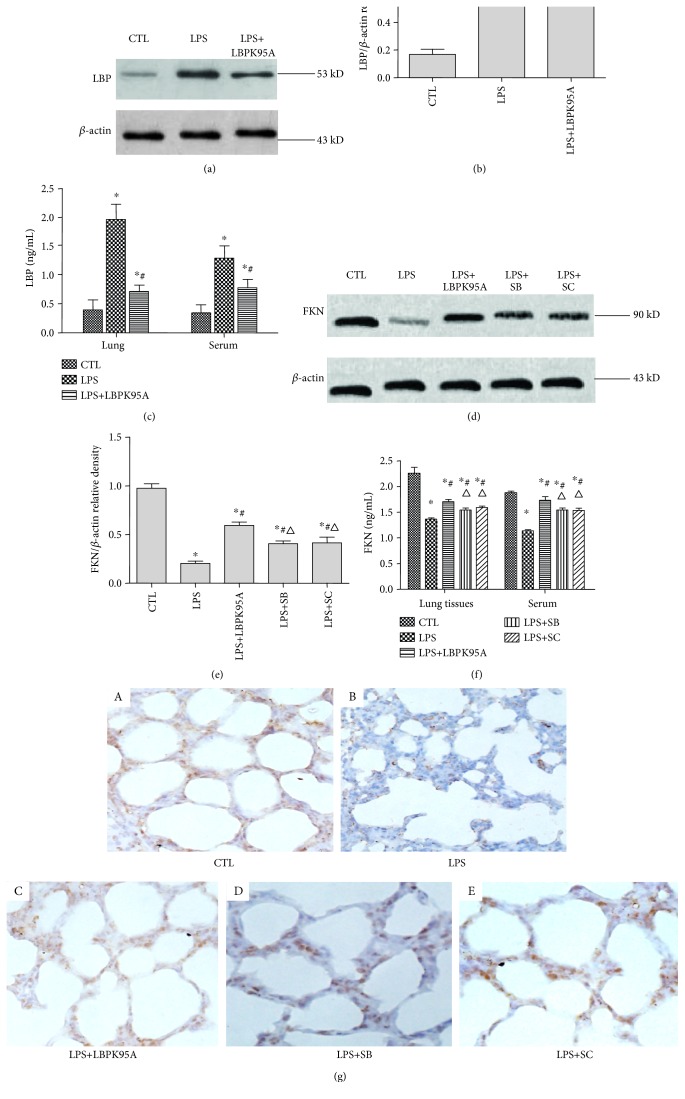
Expression of LBP and FKN protein in lung tissue homogenates and in serum of the ARDS rat model. The LBP and FKN expression in lung tissue homogenates and sera of rats with ARDS was determined by ELISA and western blotting. The results demonstrate that (a) LPS increased the levels of LBP expression significantly in both cases (*p* < 0.05 in all cases). The LBP levels of the LPS+LBPK95A group were upregulated compared with those of the control group and downregulated compared with those of the LPS group (*p* < 0.05 in all cases) ((a), (b), and (c)). (b) LPS reduced the levels of FKN expression in both cases (*p* < 0.05 in all cases). The FKN levels of the LPS+SB and LPS+SC groups are downregulated compared with those of the control group and upregulated compared with those of the LPS group (*p* < 0.05 in all cases) ((d), (e), and (f)). The FKN levels of the LPS+SB and LPS+SC groups are lower than those of the LPS+LBPK95A group (*p* < 0.05 in all cases) ((d), (e), and (f)). ∗ represents *p* < 0.05 when compared with that in the CTL group rats; # represents *p* < 0.05 when compared with that in the LPS group rats. △ represents *p* < 0.05 when compared with that in the LPS+LBPK95A rats. Immunohistochemical staining was used to determine the localization of FKN in the rat lungs. As can be seen, the major sites of FKN expression are in alveolar type I and type II epithelia. The expression of FKN protein was compared between the different groups of rats. The staining intensity and number of cells expression of FKN decreased markedly in the LPS group, when compared to those of the control group. The use of LBPK95A, SB203580, and SC-514 partly reduced the reduction of FKN seen (g). The photomicrographs are at 400x magnification.

Western blotting results show that the levels of LBP in the LPS group were 6.74 ± 4.52 times higher than those in the control group. The levels of LBP in the LPS+LPSPK95A group were 39.14 ± 9.61% lower than those in the LPS group. ELISA results also show that the levels of LBP in the LPS group were 4.66 ± 2.06 times (lung tissues) and 3.09 ± 1.15 times (sera) higher than those in the control group (*p* < 0.05, *n* = 10, in all cases). The levels of LBP in the LPS+LPSPK95A group were 63.67 ± 1.48% (lung tissues) and 39.58 ± 5.78% (sera) lower than those in the LPS group (*p* < 0.05, *n* = 10, in all cases) (Figures 7(a), 7(b), and 7(c)), indicating that LPS induced LBP expression and LBPK95A suppressed LBP expression induced by LPS. Western blotting results show that LPS reduced FKN expression by 79.68 ± 4.86%. LBPK95A, SB203580, and SC-514 increased 3.09 ± 1.15, 3.09 ± 1.15, and 3.09 ± 1.15 times of FKN levels, respectively, in lung tissue compared with that in the LPS group (*p* < 0.05, *n* = 10, in all cases) (Figures 7(d) and 7(e)). At the same time, ELISA results show that LPS reduced FKN expression in lung tissue homogenates by 37.64 ± 7.68, 38.68 ± 7.61, and 39.78 ± 3.58%, respectively. LBPK95A, SB203580, and SC-514 suppressed LPS-induced downregulation of FKN in lung tissue homogenates and sera of the ARDS rat model. FKN concentrations in the LPS+LBPK95A, LPS+SB, and LPS+SC groups were 24.55 ± 7.97, 12.73 ± 4.21, and 16.20 ± 3.01% higher in lung tissue homogenates and 53.16 ± 2.07, 37.14 ± 10.07, and 35.88 ± 20.12% higher in sera, respectively, than those in the LPS group (*p* < 0.05, *n* = 10, in all cases) (Figure 7(f)). Immunohistochemical staining shows that the FKN is expressed in alveolar type I and type II epithelia. LBPK95A, SB203580, and SC-514 suppressed the LPS-induced downregulation of FKN expression in lung tissue (Figure 7(g)).

### 3.8. LBPK95A Suppressed Phospho-p38 MAPK and Phospho-p65 Activation; SB203580 and SC-514 Suppressed Phospho-p38 MAPK and Phospho-p65 Activation, Respectively, in LPS-Induced ARDS Rats

**Figure 8:**
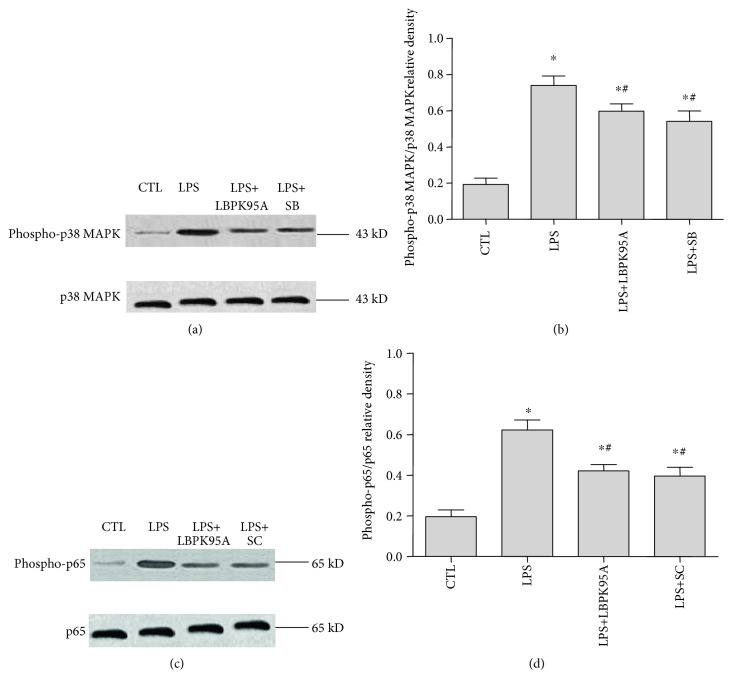
Expression of phospho-p38 MAPK and phospho-p65 in lung tissue homogenates from rats with ARDS. Western blotting was used to detect the expression of phospho-p38 MAPK and phospho-p65 in the ARDS rat model with and without inhibitors following LPS treatment. LBPK95A, SB203580, and SC-514 inhibited the LPS-induced expression of phospho-p38 MAPK (a) and phospho-p65 (b) as seen from the relative densities of phospho-p38 MAPK/*β*-actin (c) and phospho-p65/*β*-actin (d). ∗ represents *p* < 0.05 when compared with that in the control rats; # represents *p* < 0.05 when compared with that in the LPS-induced rats. △ represents *p* < 0.05 when compared with that in the LPS+LBPK95A rats.

Western blot analysis results show that the expressions of phospho-p38 MAPK and phospho-p65 in the LPS group were 3.5 ± 1.61 and 2.85 ± 1.53 times higher than those in the control group, respectively (*p* < 0.05, *n* = 10, in all cases); the expressions of phospho-p38 MAPK and phospho-p65 in the LPS+LBPK95A group were 18.14 ± 10.13% and 31.86 ± 8.47% lower than those in the LPS group, respectively. The expressions of phospho-p38 MAPK in the LPS+SB group and phospho-p65 in the LPS+SC group were 28.01 ± 12.14% and 37.38 ± 10.23% lower than the LPS group, respectively (*p* < 0.05, *n* = 10, in all cases; Figure 8). The results suggested that LPS induced the activation of phospho-p38 MAPK and phospho-p65 in the ARDS rat model. LBPK95A, SB203580, and SC-514 were able to suppress this activation.

## 4. Discussion

ARDS is a life-threatening respiratory syndrome characterized by a high incidence of pulmonary edema and acute inflammation which can result in mortality [1]. Both direct (pneumonia, aspiration, and contusion) and indirect (sepsis, trauma, and pancreatitis) lung insults could trigger the acute onset of ARDS. The pathogenic mechanisms vary depending on the initial insult. ARDS has a number of common pathological pulmonary features, such as loss of the alveolar-capillary barrier, increased permeability of alveolar epithelium and endothelium, neutrophil infiltration, and the activation of coagulation. The inflammatory cell influxes include neutrophil infiltration into the alveoli, and lung inflammatory response/cytokine production has been suggested as major contributors to the onset and progression of ARDS. LPS is a major component of Gram-negative bacteria and has been used to induce ALI/ARDS and other inflammatory diseases in several in vivo experiments [37, 38]. The use of the LPS-induced ARDS rat model is a relatively easy way to explore the mechanisms of ARDS and can provide useful biochemical information on ARDS in humans. LPS has been successfully used to establish the ARDS rat model [37, 39].

LBP is a key participant in the inflammatory response to LPS-induced infection. LBP is an enhancer of cellular LPS responsiveness via its unique property to transfer LPS to the CD14/TLR4 receptor complex on immune cells [40]. It has been shown that pulmonary LBP is upregulated following LPS-mediated injury, and circulating LBP levels are increased in several infectious diseases, such as septic shock and acute lung inflammation [41]. In this study, we showed that LPS increased the LBP mRNA and protein expression in A549 cells and the LPS-induced ARDS rats. LBP gene overexpression further enhanced the induction of LPS on LBP. Inhibiting LBP by LBP gene silencing or using an LBP blockade peptide decreased the induction of LPS on LBP. LBP interacts with membrane-bound or soluble CD14, LPS, and toll-like receptor 4 to initiate cellular production of proinflammatory cytokines, immune cell recruitment, and endotoxin clearance [42]. The extent of NF-*κ*B activation and TNF-*α* expression was significantly increased in the cells treated with LBP and LPS together, suggesting that LBP could bind to free LPS to form an LPS/LBP complex, which would then deliver LBP to CD14 on the surfaces of monocytes and macrophages in order to amplify the inflammatory effects of LPS. However, LPS/LBP complex-induced NF-*κ*B activation and TNF-*α* expression were significantly inhibited by LBP inhibitory polypeptides by blocking the LBP site from binding to CD14, thus preventing LPS from binding to CD14 via LBP. This probably interferes with the LBP/CD14 sensitizing system and, thereby, inhibits LPS-induced release of inflammatory factors [14]. The LBP blockade peptide, LBPK95A, originated from the LBP protein sequence 86–99, with a mutation of 95 lysine to alanine, and this was able to decrease upregulated LBP that had been induced by LPS and block LBP-LPS interactions. Thus, this was able to neutralize efficiently LPS toxicity in vivo and prevent mice mortality by Gram-negative bacteremia which further supports the notion that lethal inflammatory responses toward endotoxins could be effectively reduced or abrogated by inhibiting the LBP-LPS interaction [43]. LPS binds CD14/TLR4 receptor complex on the surface of epithelial cells, activates p38 MAPK and NF-*κ*B signal transduction pathways through the myeloid differentiation factor 88- (MyD88-) dependent pathway, and then induces the expression of many inflammatory markers [44, 45]. p38 MAPK and NF-*κ*B pathways have been reported to be involved in the release of proinflammatory mediators in ARDS. In vivo, treatment with SB203580 substantially inhibited LPS-induced neutrophil recruitment into the lungs and changes in lung injury parameters, such as total protein content in BAL fluid and apoptosis of neutrophils and macrophages [46, 47]. Inhibition of p38 MAPK decreased injury to the lung through attenuated production of TNF-*α* and nitric oxide in the rat model of pancreatitis-induced ARDS [48]. SB203580 is a classical inhibitor of p38 MAPK that prevented the activation and phosphorylation of MAPKAP kinase-2 and the phosphorylation of hsp27 by cellular stresses, LPS, and IL-1 [49]. NF-*κ*B is an important transcription factor and mediates the expression of many inflammatory cytokines and cell adhesion molecules during inflammation. The transcriptional activity of NF-*κ*B depends on the posttranslational modification of p65. SC-514 is a cell-permeable, potent, and selective ATP competitive inhibitor of NF-*κ*B-2 (I*κκ*-2). I*κκ*-2 inhibition by SC-514 demonstrates a decreased level of IKK-2 phosphorylation/degradation, and diminished LPS induces p65 translocation into the nucleus [50]. In this study, we show that LPS not only induced phospho-p38 MAPK and phospho-p65 expression but also promoted phospho-p38 MAPK and phospho-p65 transfer to the nucleus and enhanced interaction between LBP and phospho-p38 MAPK and LBP and phospho-p65, respectively. LBP gene overexpression and LBP gene silencing strengthened and weakened the effect induced by LPS, respectively. The LBP blockade peptide, LBPK95A, inhibited phospho-p38 MAPK and phospho-p65 increases in lung tissues of the LPS-induced ARDS rats. These results suggest that LPS induced p38 MAPK and NF-*κ*B signaling pathways activation by LBP.

FKN has been recognized as a proinflammatory cytokine. FKN contributed to several inflammatory disorders, such as acute necrotizing pancreatitis, tuberculosis, and sepsis [16, 51, 52]. FKN binds to its receptor CX3CR1, promoting neutrophil adhesion to epithelial cells, migration and tissue invasion, production of oxygen free radicals, and general expansion of the inflammatory reaction [53]. FKN can be induced by inflammatory mediators such as IFN-*γ* and TNF-*α* but is reduced by some inflammatory mediators such as TGF-*β* [54]. FKN has been reported to be decreased in some inflammatory diseases, such as urogenital tract inflammation, which therefore implies an anti-inflammatory effect of FKN [55]. Recent research has shown that FKN attenuated the LPS-induced production of IL-1, IL-6, and TNF-*α* by rat and mouse microglia, which are phagocytotic cells that are responsible for cytokine production in the CNS [56]. Exogenous FKN injected into the mice did not increase the expression of TNF-*α*, while the use of anti-FKN antibodies enhanced the inflammatory effect of LPS [57]. However, the anti-FKN antibody alone demonstrated similar toxicity to LPS and, in combination with anti-FKN antibody, was able to enhance LPS toxicity.

In this study, we showed that LPS increased the LBP mRNA and protein expression and reduced the FKN production both in cultured A549 cells and in an ARDS rat model. Overexpression of LBP gene by transfection with LBP plasmid further decreased LPS-induced downregulation of FKN mRNA and protein expression whereas silencing of the LBP gene inhibited LPS-induced downregulation of FKN. SB203580 and SC-514 pretreated A549 cells resulted in the inhibition of p38 MAPK and p65 NF-*κ*B transduction, respectively, and the suppressive effect on the LPS-induced reduction of FKN. These results suggest that both p38 MAPK and NF-*κ*B are involved in the release of FKN in A549 cells. LBP increased LPS-induced reduction of FKN by activating both p38 MAPK and p65 NF-*κ*B signaling pathways in vitro. Immunohistochemical staining results indicated that pretreatment of the ARDS rats with LBPK95A, SB203580, and SC-514 reduced lung injury caused by LPS. The results of both in vitro and in vivo experiments demonstrated that LPS reduced FKN expression and caused rat lung injury and that LBPK95A, SB203580, and SC-514 inhibited these effects and reduced lung injury in a p38 MAPK- and NF-*κ*B-dependent manner. These results suggest an anti-inflammatory and protective effect of FKN in ARDS. FKN could therefore be considered as a potential therapeutic target for regulating the inflammatory response in ARDS.

In this study, we show that LPS inhibited FKN expression through p38 MAPK and NF-*κ*B signaling pathways, but some studies have demonstrated an increased expression of FKN in chronic respiratory disease such as asthma and chronic obstructive pulmonary disease through p38 MAPK or NF-*κ*B signaling pathways. Previous reports have shown that FKN mainly upregulated expression in the airway tissues such as airway epithelium, submucosa, and smooth muscle of chronic respiratory disease, but we show that LPS inhibited FKN expression in lung tissues especially in alveolar epithelial cells of acute inflammatory lung disease. Some studies show that TNF-*α*-stimulated VSMC fractalkine mRNA and protein expression are attenuated by pharmacologic inhibitors of PKC and p42/44 MAPK kinase, but not p38 MAPK, indicating that the intracellular signals mediating TNF-*α*-stimulated fractalkine expression involve the activation of PKC and p42/44 MAPK, rather than p38 MAPK pathway [31]. In most studies where FKN is shown to be increased, NF-*κ*B is activated by a classical heterodimer of NF-*κ*B (p65-p50) formation [58]. However, in this study, FKN decreased when induced by LPS and this was inhibited by SC-514 which is a selective ATP competitive inhibitor of NF-*κ*B-2 (I*κκ*-2, p52) and it is able to block p65-p52 heterodimers but not p65-p50.

Although we were able to show the mechanism of LBP regulation of FKN and its related signaling pathways in a cultured cell line and in an ARDS rat model, further research is required to investigate the anti-inflammatory effects of FKN and its potential role in ARDS treatment.

## 5. Conclusions

In conclusion, the data presented here show that LBP further decreased LPS-induced reduction of FKN and LPS-induced lung injury and these effects can be prevented by transfection with LBP shRNA plasmid or using LBP inhibitory peptide and inhibition of the activation of p38 MAPK and NF-*κ*B signaling transduction pathways, suggesting that LBP downregulates FKN expression through the activation of p38 MAPK and NF-*κ*B in the progression of ARDS.

## Figures and Tables

**Table 1:** The primer sequences and Genbank numbers for the primers used.

Gene	Genbank number	Sense primer	Antisense primer
LBP (human)	NM_004139	5′-tggctgttgaacctcttcca-3′	5′-tgtcggcgaaactgtcaatc-3′
LBP (rat)	NM_017208	5′-agcggggacttcaagatcaa-3′	5′-ggatctgcgcactttccatt-3′
FKN (human)	NM_002996	5′-aactcgaatggcggcacctt-3′	5′-atgaattactaccacagctccg-3′
FKN (rat)	NM_134455	5-agcctcagagcactggaat-3′	5′-ggtggacgattgagtagatag-3′
GAPDH (human)	NM_001289746	5′-ccacccatggcaaattccatggca-3′	5′-tctagacggcaggtcaggtccacc-3′
GAPDH (rat)	NM_017008	5′-gatttggccgtatcggac-3′	5′-gaagacgccagtagactc-3′

## Abbreviations

ARDS: Acute respiratory distress syndrome
BALF: Bronchoalveolar lavage fluid
Co-IP: Coimmunoprecipitation
FKN: Fractalkine
GAPDH: Glyceraldehyde-3-phosphate dehydrogenase
LBP: LPS-binding protein
LPS: Lipopolysaccharide
MAPK: Mitogen-activated protein kinases
MPO: Myeloperoxidase
NF-*κ*B: Nuclear factor-*κ*B
TNF-*α*: Tumor necrosis factor-*α*.
